# Synchronous malignancies of the gall bladder and common bile duct: a case report

**DOI:** 10.1186/s12957-016-0848-y

**Published:** 2016-04-08

**Authors:** Srinivas Kodaganur, Ishwar R. Hosamani

**Affiliations:** Department of General Surgery, KIMS, Hubli, Karnataka 580022 India

**Keywords:** Synchronous malignancies, Periampullary carcinoma, Gall bladder carcinoma, Pancreaticoduodenectomy, Anamolous pancreatic-bile duct junction (APBDJ)

## Abstract

**Background:**

Synchronous malignancies of the gall bladder and common bile duct are a rare entity. Much of our knowledge on this topic comes from Japanese literature. Most of the synchronous carcinomas described in Japanese literature are associated with the presence of an anomalous pancreatic-bile duct junction (APBDJ).

**Case presentation:**

We report a case of synchronous malignancy of the extrahepatic biliary tree involving the fundus of the gall bladder and the intrapancreatic portion of the common bile duct (CBD). A 50-year-old female patient presented to us with clinical features of obstructive jaundice and on radiological evaluation was diagnosed to have a periampullary carcinoma; the patient underwent a pancreaticoduodenectomy, and histopathological examination revealed adenocarcinoma of the gall bladder and the intrapancreatic portion of the CBD.

**Conclusions:**

Synchronous malignancies have been rarely reported from the Indian subcontinent; therefore, it is essential for the clinician as well as the pathologist to maintain a high index of suspicion while evaluating such lesions and to look for the presence of an anamolous pancreatic-bile duct junction whenever indicated.

## Background

Synchronous malignancies of the gall bladder and common bile duct are a rare entity. Much of our knowledge on this topic comes from Japanese literature [1, 2].

Most of the synchronous carcinomas described in Japanese literature are associated with the presence of an anomalous pancreatic-bile duct junction (APBDJ) [1, 2]. However, Kurasoki et al. have shown that the presence of APBDJ is not an absolute necessity for the development of synchronous malignancies of the biliary tree [3].

The data from the rest of the world is sparse. Shukla et al. from India have published an original article describing four cases of simultaneous malignancies of the gall bladder and common bile duct of which two are synchronous.

Here, we report a synchronous malignancy of the gall bladder and common bile duct, its diagnosis, and successful management.

## Case presentation

A 50-year-old lady presented with complaints of pain abdomen and jaundice for one and a half months; she also complained of intermittent type of fever in the last 1 week. Physical examination revealed presence of icterus and a palpable tender globular mass in the right hypochondrium measuring 3 × 3 cm. A clinical diagnosis of obstructive jaundice secondary to periampullary carcinoma was made.

Ultrasonographic examination of the abdomen and pelvis revealed dilatation of the intrahepatic biliary radicles (IHBR), with multiple freely mobile gall stones and a dilated common bile duct (CBD) measuring 1.8 cm with sudden narrowing at its distal end.

**Fig. 1:**
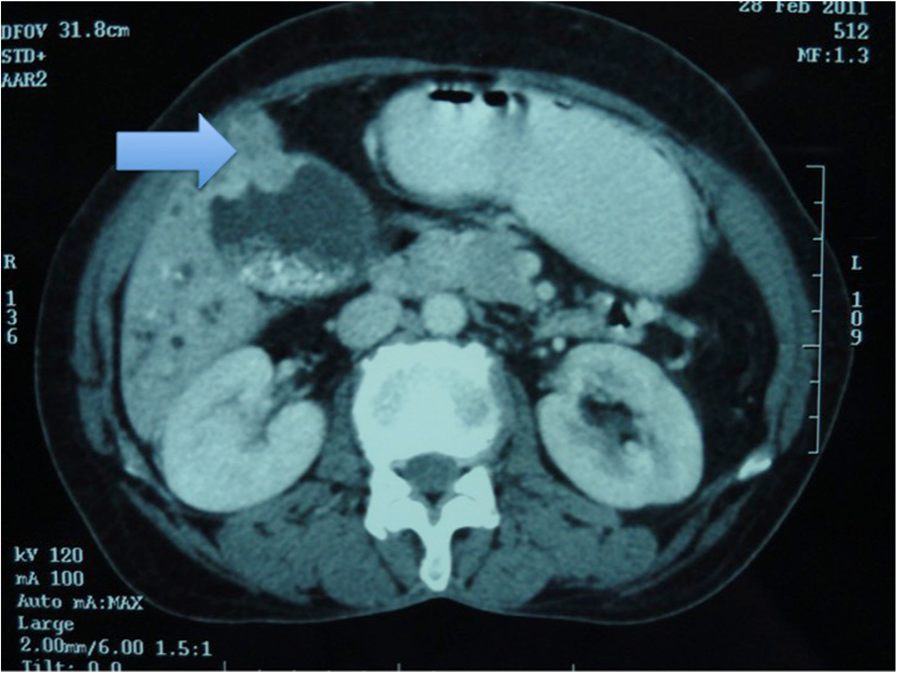
CECT of the abdomen showing thickening of the gall bladder wall at the fundus and multiple gallstones

CT scan showed moderate dilatation of the extra and intrahepatic biliary radicles, cholelithiasis with suspicious thickening of the gall bladder wall at the fundus, and a distal CBD stricture. There was no evidence of any distant metastasis (Fig. 1).

A preoperative diagnosis of periampullary carcinoma with chronic cholecystitis was made, and the patient planned for a pancreaticoduodenectomy (Whipple’s procedure) after optimization.

The patient underwent a classical Whipple’s procedure; table examination of the resected specimen revealed multiple gall stones in the gall bladder with area of mucosal thickening at the fundus and an irregular circumferential growth involving the intrapancreatic portion of the CBD.

Postoperative period was uneventful, and the patient recovered satisfactorily.

**Fig. 2:**
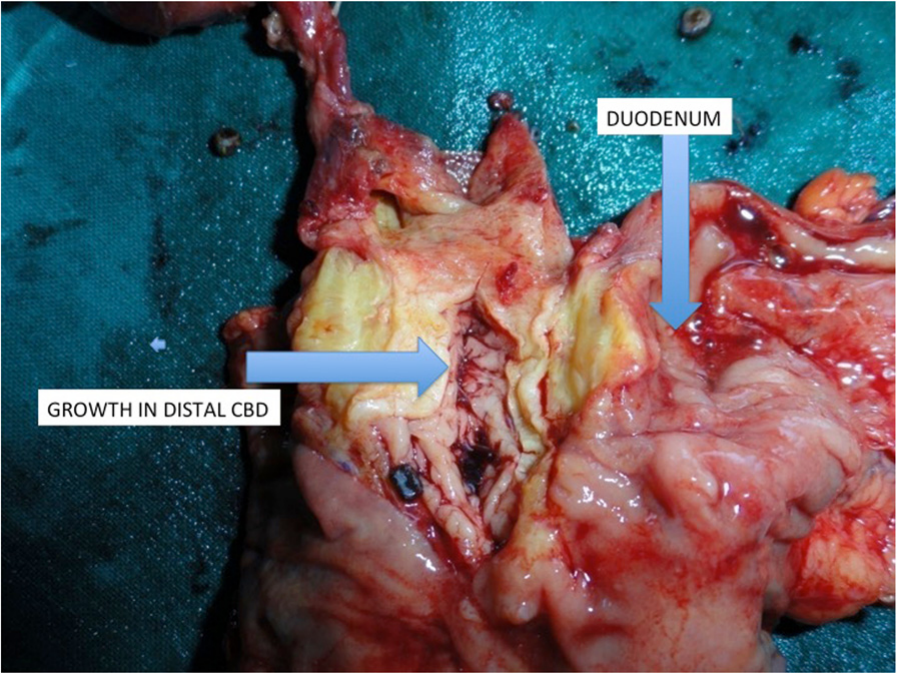
Postoperative specimen with *arrows* pointing towards duodenum and growth in the intrapancreatic portion of the CBD

**Fig. 3:**
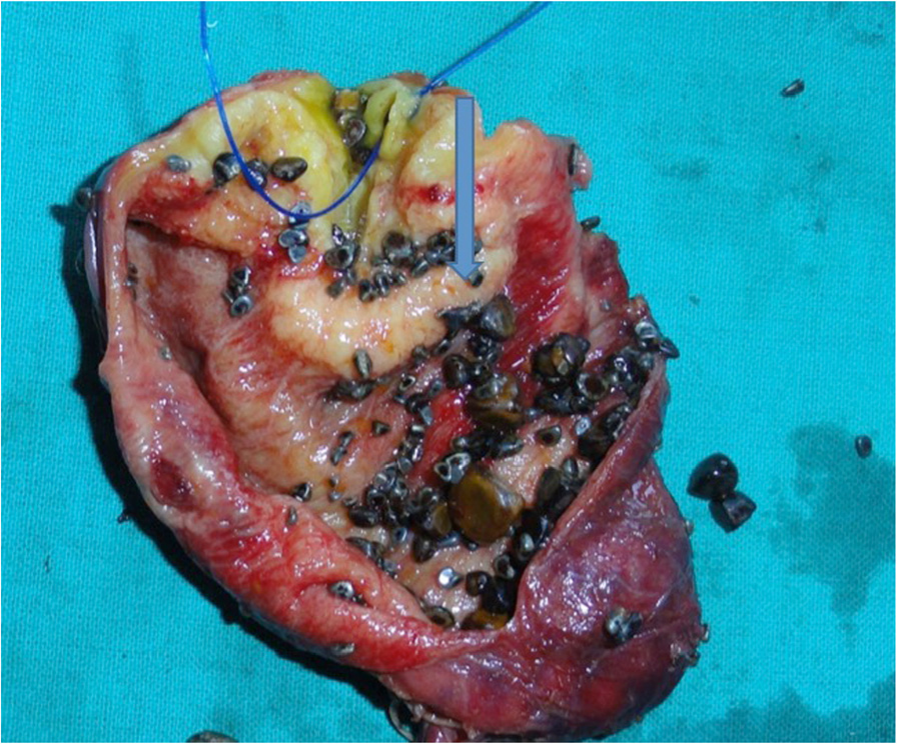
Postoperative specimen with an *arrow* pointing at the thickening of the gall bladder wall at the fundus

Histopathological examination of the resected specimen (Figs. 2 and 3) revealed thickening of the gall bladder wall at the fundus with multiple small calculi, the largest measuring 1.4 cm (Fig. 3). Sections studied from the area of thickening showed features suggestive of adenocarcinoma limited to the mucosa.

Microscopic sections obtained from the distal CBD region showed adenocarcinoma of the common bile duct with invasion into the muscular wall of the duodenum.

Sections studied from the resected lymph nodes were free of tumor cells.

A pathological staging of pT1aN0M0 for adenocarcinoma of the gall bladder and pT2N0M0 for adenocarcinoma of the distal CBD was made, and the patient was not subjected to any adjuvant therapy.

The patient was disease-free 2 years after surgery.

### Discussion

The data from Japanese literature suggest that synchronous malignancies of the biliary tree are not as rare as they were thought to be; its incidence is found to be as high as 5.0 to 7.4 %. The reason for their earlier reported low incidence was probably due to inadequate sampling of the gall bladder specimen after resection for extrahepatic tumors [3].

When simultaneous malignancies of the gall bladder and common bile duct are discovered, it is important to distinguish between synchronous primary malignancies and secondary deposits. Warren et al. and Gertsch et al. have described certain criteria to differentiate synchronous primaries from malignant deposits, and they include (I) lack of anatomical continuity between the two tumors, (II) a growth pattern typical of a primary tumor, and (III) clear histological differences between the two tumors [4, 5]. In our patient, all the three criteria have been fulfilled; the adenocarcinoma of the gall bladder was secondary to chronic cholecystitis and at an early stage (pT1aN0M0), whereas the periampullary lesion was more advanced (pT2N0M0); both were labeled as primary adenocarcinomas by the pathologist as they had a growth pattern typical of a primary tumor.

APBDJ is one of the main etiological factors for synchronous malignancies of the biliary tree [1, 2]. In a study by Hara et al., 77 patients diagnosed with APBDJ were found to have a higher cellular proliferation rate of the biliary epithelium compared to matched controls thereby increasing the risk of developing malignancy; the same authors recommend that the extrahepatic bile ducts be prophylactically excised in all patients with APBDJ [6]. In our patient, the pancreatic-bile duct junction could not be evaluated due to the presence of the periampullary carcinoma.

As we were unable to diagnose the gall bladder carcinoma preoperatively, the presence of a synchronous malignancy was a histological surprise. But fortunately, as the gall bladder carcinoma was not involving the muscularis, no further treatment was needed and an R0 resection was achieved with a classical Whipple’s procedure.

Shukla et al. have proposed various pathways for development of synchronous malignancies of the biliary tree and have reported four cases through which they explain the various possible pathways of development of simultaneous malignancies of the biliary tree; our case is similar to the first case described in the article in which the authors hypothesize that these are truly synchronous malignancies and sometimes may be associated with APBDJ but can occur even in the presence of a normal pancreatic-bile duct junction [7].

## Conclusions

Synchronous malignancies of the biliary tree are becoming more common in current hepatobiliary practice, and it is essential for the clinician as well as the pathologist to maintain a high index of suspicion while evaluating such lesions and to look for the presence of an anamolous pancreatic-bile duct junction whenever indicated.

Informed consent was taken for publications of photographs in a scientific journal according to the guidelines of the Declaration of Helsinki and its later amendments.
